# Beneficial Effect of Paeonol on Antibiotic-Associated Inflammatory Response in Mice with Diarrhea

**DOI:** 10.3390/biom12111634

**Published:** 2022-11-03

**Authors:** Bohyung Kang, Do Hwi Park, Myong Jin Lee, Chan-Yong Jeon, Ki Sung Kang, You-Kyung Choi

**Affiliations:** College of Korean Medicine, Gachon University, Seongnam 13120, Korea

**Keywords:** herbal medicine, moutan radicis cortex, gut microbiota, anti-inflammatory

## Abstract

Diarrhea is a common adverse effect of antibiotics particularly that acts on anaerobes. Moutan Radicis Cortex (MRC) is an herbal medicine used for its anti-inflammatory and antibacterial actions. The purpose of this study was to analyze the active components of MRC to determine their effect on antibiotic-associated diarrhea (AAD) and anti-inflammatory effects. Of the various components of MRC, seven compounds (gallic acid, oxypaeoniflorin, paeoniflorin, ethyl gallate, benzoic acid, benzoylpaeoniflorin, paeonol) were identified and assessed for anti-inflammation effects. Paeonol was found to effectively reduce nitric oxide production and levels of IL-6 and TNF-α in a concentration-dependent manner. Paeonol also effectively reduced the mRNA expression level of IL-6, IL-1β, and TNF-α. Western blotting analysis confirmed the reduction of COX-2 and NF-κB levels; p-p38 MAPK levels increased in the presence of a low concentration (25 μM) of paeonol but decreased in the presence of a high concentration (50 μM). In the mouse model of lincomycin-induced AAD, all experimental groups treated with paeonol (25, 50, and 100 mg/kg concentrations) showed diminished diarrhea status scores. Finally, the expression levels of TNF-α and IL-4 were reduced compared with those in the control group. Therefore, paeonol may have active compounds of MRC to alleviate the diarrhea symptoms of AAD and reduce inflammatory mediators. Other components of the MRC extract could contribute to its known anti-inflammatory and antibacterial activity and should be tested for their possible activity.

## 1. Introduction

Inappropriate use of antibiotics can result in long-term complications, such as obesity, diabetes, inflammatory bowel disease, allergies, and asthma [1]. Antibiotics may increase susceptibility to infection and disease by killing beneficial and harmful bacteria [1]. When antibiotics kill beneficial bacteria, they become more susceptible to infection and disease and can be targets for harmful bacteria. [2]. Antibiotic-associated diarrhea (AAD) is mainly caused by alterations in the immune environment of the gut; antibiotics can break down the colonization resistance of the intestinal flora that prevents the growth of pathogens and change the content of fatty acids in the intestine or increase the amount of carbohydrates that cannot be absorbed. Furthermore, AAD is closely related to non-intestinal conditions, such as diabetes and allergies [3]. Clinical manifestations of AAD range from diarrhea with frequent loose stools to colitis, which can develop into a serious disease. In severe cases, AAD refers to *Clostridium difficile* diarrhea (*C. difficile* associated diarrhea, CDAD).

Currently, clinical trials recommend treatment with metronidazole or vancomycin supplemented with pro-biotics that replenish microorganisms and activate the intestinal flora. Other methods are being tested, including immunotherapy [4]. It is reported that the frequency of the disease occurrence is increasing yearly; in the United States, since 2006, over 500,000 CDAD cases have been occurring annually, with concomitant annual medical expenses of over $1 billion [5]. Hence, in addition to proper antibiotic usage control and development of new antibiotics, there is a growing interest in treatments using complementary and alternative medicines, such as herbal medicines, probiotics, and fecal transplants [6].

Therefore, it is of utmost importance to identify the scale of antibiotic use, reduce indiscriminate use, and develop methods to treat side effects and alternatives to antibiotics [7]. Although several clinical trials in oriental medicine such as Hyangsayukgunja-tang have demonstrated the potential application of herbal medicines to treat AAD [3,8], mechanism of action and active compound of herbal medicines have not been identified yet. In the recent systematic review on traditional Chinese herbal medicine treatment for AAD, evidence of treatment efficacy for AAD is encouraging, but not conclusive because of the low methodological qualities and diversity of treatment prescriptions [8]. In our ongoing project on the quality evaluation of various herbal medicine prescriptions, we found that anti-inflammatory action on MRC can be helpful for AAD based on several screening results. Therefore, a study was conducted to elucidate the active compound for diarrhea recovery in the ADD model of MRC.

Moutan Radicis Cortex (MRC) is an herbal medicine used in herbal prescriptions such as Palmijihwanghwan, Yukmijihwangtang, Gagampalmihwan and Gamishingihwan, and is known for its anti-inflammatory and antibacterial actions. MRC contains several biologically active compounds such as paeoniflorin, oxypaeoniflorin, benzoylpaeoniflorin, albiflorin, which bind to sugar, and paeoniflorigenone and pallotanin, which do not bind to sugar [9]. In this study, a total of seven compounds, including gallic acid, oxypaeoniflorin, paeoniflorin, ethyl gallate, benzoic acid, benzoylpaeoniflorin, and paeonol, were quantitatively analyzed and examined for anti-inflammatory effects. Paenol displayed good anti-inflammatory properties and selected to confirm its efficacy in treating AAD. We examined the changes in the diarrhea status, body weight, and cytokine levels by paeonol treatment to confirm its effect. These results might offer some new data on a natural product for the treatment of AAD.

## 2. Materials and Methods

### 2.1. Plant Material and Reagents

MRC collected in Danyang (Korea) was purchased from Medical Herbs (Ulsan, Korea) and was identified by Goya Choi, Korea Institute of Oriental Medicine (Naju, Korea). A voucher specimen (MRC-42) was deposited at the College of Korean Medicine, Gachon University (Seongnam, Korea). Benzoic acid (99.9%), gallic acid (99.0%), and paeonol (99.9%) were purchased from Merck KgaA (Darmstadt, Germany); benzoylpaeoniflorin (98.0%), oxypaeoniflorin (98.0%), and paeoniflorin (99.4%) were purchased from Shanghai Sunny Biotech Co., Ltd. (Shanghai, China); ethyl gallate (99.5%) was purchased from Tokyo Chemical Industry Co., Ltd. (Tokyo, Japan). Methanol, acetonitrile, and water, used as solvents for HPLC analysis, were purchased from J.T. Baker (Phillipsburg, NJ, USA) and were of HPLC grade. Formic acid (American Chemical Society reagent-grade, ≥98.0%) for mobile phase preparation was obtained from Merck (Darmstadt, Germany).

### 2.2. Preparation of 70% Ethanol Extract of MRC

Dried MRC (700 g) was extracted three times using 70% ethanol (7.0 L) for 1 h at room temperature using a Branson 8520 sonicator (Danbury, CT, USA). The extracted solution was washed with Whatman filter paper No. 2 (150 mm Ø; Maidstone, Kent, UK) and concentrated to remove ethanol using a Büchi R-210 rotary evaporator (Flawil, Switzerland). The extracted sample was freeze-dried using a BioBase freeze dryer (FD-5525L; Ilshin, Dongducheon, Korea). The amount of freeze-dried 70% ethanol extract of MRC was 146.89 g (yield 21.0%).

### 2.3. HPLC Analysis of the MRC Compounds

Simultaneous measurements of the seven compounds of MRC, gallic acid, oxypaeoniflorin, paeoniflorin, ethyl gallate, benzoic acid, benzoylpaeoniflorin, and paeonol were performed by a photodiode array (PDA) detector and LabSolution software (Version 5.53, SP3) for data collection and conversion, and quantified using Shimadzu Prominence LC-20A Series (Kyoto, Japan). Efficient separation of sample was performed with a reverse-phase SunFire C18 analytical column (4.6 × 250 mm, 5 μm; Waters, Torrance, CA, USA) maintained at 30 °C at a flow rate of 1.0 mL/min. An aliquot of samples were injected with an auto sampler SIL-20A, and the HPLC injection volume was 10 μL. The mobile phase consisted of 0.1% formic acid in distilled water (solvent A) and 0.1% formic acid in acetonitrile (solvent B). The analysis was performed under the following gradient elution conditions: 0–40 min, 5–40% B; 40–45 min, 40% B; and 45–50 min, 40–5% B. The flow rate of the mobile phase was set at 0.8 mL/min, and the injection volume of the standard and test solutions was 10 μL each. For the simultaneous analysis of the seven marker components of MRC, 100.0 mg of freeze-dried MRC extract was dissolved in 10 mL of 70% methanol and sonicated for 60 min. Quantification of oxypaeoniflorin, paoniflorin, and paeonol was performed by pure standard injections. All sample solutions were filtered using a 0.2 μm syringe filter (Pall Life Sciences, Ann Arbor, MI, USA) prior to analysis. Quantification of each analyte was performed with standard calibration curves, as shown in Table 1. Linear range, regression equation, and coefficient of determination (r^2^) are also presented in Table 1.

### 2.4. Cell Culture

Murine macrophage cell line RAW 264.7 (American Type Culture Collection, Rockville, MD, USA) was cultured in Dulbecco’s modified Eagle’s medium (DMEM; Manassas, VA, USA), containing 4 mM L-glutamine, antibiotics (1% penicillin/streptomycin), and 10% fetal bovine serum (FBS), in a humidified air environment of 5% CO_2_ at 37 °C.

### 2.5. Cell Viability Assay

Compound dissolved well in DMSO stock solution (5 mM), and diluted with cell culture media for the cell experiments. RAW 264.7 cells (3 × 10^4^ cells/well) were exposed to different concentrations (6.3, 12.5, 25, and 50 μM) of 7 compounds of MRC that were purchased from Shanghai Sunny Biotech and Ez-Cytox solution (Daeil Lab Service Co., Seoul, Korea) at 37 °C for 24 h. Optical density (OD) at 450 nm was determined using a microplate spectrophotometer (PowerWave XS; Bio-Tek Instruments, Winooski, VT, USA).

### 2.6. Measurement of Nitric Oxide (NO) Production Levels

An NO assay was performed to assess inhibition of NO secretion. The cells were treated with 7 compounds of MRC that were purchased from Shanghai Sunny Biotech for 2 h followed by stimulation with 100 ng/mL lipopolysaccharide (LPS) from *Escherichia coli* O26:B6 (Sigma-Aldrich, St. Louis, MO, USA) for 22 h. The cell culture supernatants were subsequently incubated with the same amount of Griess reagent. The NO concentration was evaluated by measuring OD at 550 nm using a micro-plate spectrophotometer.

### 2.7. Measurement of Cytokine Production Levels

The cells were incubated with various concentrations of paeonol (Shanghai Sunny Biotech Co., Ltd.) for 2 h and then treated with 100 ng/mL of LPS for 22 h. Then, the levels of tumor necrosis factor-alpha (TNF-α) and interleukin 6 (IL-6) were measured using a sandwich enzyme-linked immunosorbent assay (ELISA) kit according to the manufacturer’s instructions. The experiments were performed in triplicate.

### 2.8. Western Blotting Analysis

After incubation of cells (8 × 10^5^ cells/well) in 60-mm dishes, the cells were treated with various concentrations of paeonol for 2 h, followed by treatment with LPS for 22 h. Whole-cell extracts were prepared using RIPA buffer (Cell Signaling, Danvers, MA, USA) supplemented with a 1× protease inhibitor cocktail and 1 mM phenylmethylsulfonyl fluoride according to the manufacturer’s instructions. Proteins were separated by electrophoresis using precast 4–15% Mini-PROTEAN TGX gels (Bio-Rad, Hercules, CA, USA), transferred to polyvinylidene fluoride membranes The membranes were incubated with the primary antibodies anti-iNOS (1:1000), anti-Cox-2 (1:1000), anti-NF-κB (1:1000), anti-Lamin B1 (1:1000), anti-p-JNK (1:1000), anti-JNK (1:1000), anti-p-ERK (1:1000), anti-ERK (1:1000), anti-p-p38 (1:1000), anti-p38 (1:1000), and anti-GAPDH (1:1000) overnight at 4 °C. After three washings, the blots were incubated with secondary antibodies conjugated with horse peroxidase (1:10,000) for 1 h at RT. The used antibodies were purchased from CST (Cell Signaling Technology). Bound antibodies were visualized using ECL Advance Western Blotting Detection Rea-gents (GE Healthcare, Buckinghamshire, UK) and an LAS 4000 imaging system (Fujifilm, Tokyo, Japan).

### 2.9. Gene Expression Analysis Using Real-Time Polymerase Chain Reaction (Quantitative qPCR)

RAW 264.7 macrophages were cultured in a 6-well plate for 24 h in DMEM containing 10% FBS at a concentration of 5 × 10^5^ cells/well. The cells were treated with paeonol (Shanghai Sunny Biotech Co., Ltd.) for 2 h, followed by LPS (100 ng/mL) stimulation for 4 h. The cultured cells were then homogenized with 0.35 mL of RNA extraction and lysis buffer (RNeasy mini kit; Qiagen, Hilden, Germany), followed by total RNA purification according to the manufacturer’s protocol. Total RNA was reverse-transcribed into cDNA using AccuPower CycleScript RT premix (dT18; Bioneer, Daejeon, Korea) according to the manufacturer’s protocol. The PCR amplification of each cDNA template was performed using the QuantStudio 3 Real-time PCR system with Accupower 2xGreen/star qPCR master mix (Bioneer). The primers were used as follows: for IL-6, 5’-GGTACATCCTCGACGGCATCT-3’ and 5’-GT GCCTCTTTGCTGCTTTCAC-3’; for IL-1β, 5’- CCGGGACTCACAGCAAAA-3’ and 5’-GGACATGGAGAACACCACTTG-3’; for TNF-α, 5’-CAGAGGGCCTGTACCTCATC-3’ and 5’-GGAAGACCCCTCCCAGATAG-3’and for GAPDH, 5’-TGCTGAGTATGTCGTGGA GT-3’ and 5’-GTTCACACCCATCACAAACA-3’. The mRNA expression levels of IL-6, interleukin 1 (IL-1), and TNF-α were calculated using the 2^−∆∆CT^ method and normalized to those of 18 s.

### 2.10. Immunocytochemistry

RAW 264.7 cells were fixed with 4% formaldehyde at room temperature for 20 min and washed with PBS containing 5% normal goat serum. The fixed cells were first incubated with anti-nuclear factor-kappa B (NF-κB) at 4 °C overnight and then with the secondary antibody (anti-rabbit IgG, Alexa Fluor 488 conjugate) for 1 h. Staining was visual-ized with ProLong Gold Antifade with 4′, 6-diamidino-2-phenylindole (DAPI) for 10 min and observed under a fluorescence microscope.

### 2.11. In Vivo AAD Model Generation and Treatment Experiments

**Figure 1:**
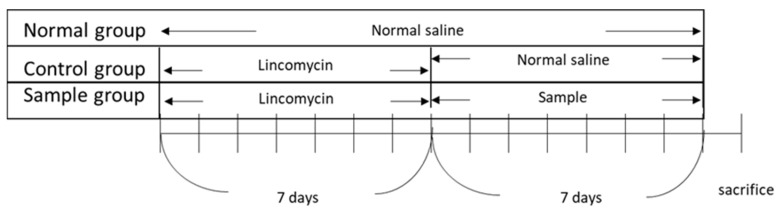
Schematic of the in vivo experiment to induce an antibiotic-associated diarrhea model in BALB/c mice.

The in vivo experiments were approved by the Institutional Animal Care and Use Committee (Internal Review Board deliberation number: KBIO-IACUC-2018-106). All procedures were conducted in strict accordance with the legislation on the use and care of laboratory animals. BALB/c mice (6 weeks old), with an average body weight of 20–21 g, supplied by Daehan Biolink (Chungcheongbuk-do, Korea), were used in the experiment. The animals were maintained in a room under standard laboratory conditions with a 12:12 h light-dark cycle, a constant temperature of 22 ± 2 °C, and 55 ± 10% humidity. At the end of the experiments, the animals were anesthetized and euthanized. All efforts were made to minimize suffering. After 7 d of acclimatization, five groups (*n* = 5/group) were tested: control group, untreated group (normal), and three experimental groups (paeonol concentrations of 25, 50, and 100 mg/kg). To establish the AAD model, lincomycin was orally administered to the animals at a dose of 3 g/kg twice daily (8 h apart) for 7 days. The untreated group (normal group) was orally administered the same amount of physiological saline without lincomycin. After developing the AAD model, a 10 mL/kg sample (paeonol) was administered to the experimental group (sample group) twice a day, while the control group was administered saline twice daily. Body weight, water intake, and diarrhea (diarrheal status score) were assessed for all groups. Diarrheal status scores were obtained by checking for diarrhea daily using a four-grade system. Scoring criteria accounted for changes in stool consistency and humidity, as follows: 0—normal stool consistency and dryness; 1—wet stools; 2—pasty stools; 3—very soft stools with traces of blood; 4—watery stools with visible rectal bleeding. At the end of the experiment, blood was collected from the hearts of each group and centrifuged (1500× *g*, 10 min) to obtain the serum. Thereafter, serum cytokine concentrations were measured using the BD OptEIA™ Rat IL-4 ELISA Set and the Rat TNF (Mono/Mono) ELISA Set. Figure 1 illustrates the timeline of the in vivo experiments.

### 2.12. Statistical Analysis

Assays were performed in triplicate and repeated at least thrice. Data are presented as mean ± standard deviation (SD). Statistical significance was determined using a one-way analysis of variance (ANOVA) and multiple comparisons with Bonferroni correction. Statistical significance was set at *p*-value < 0.05. Analyses were performed using SPSS Statistics ver. 19.0 (SPSS Inc., Chicago, IL, USA).

## 3. Results

### 3.1. Quantitative Analysis of MRC Extract Components

**Figure 2:**
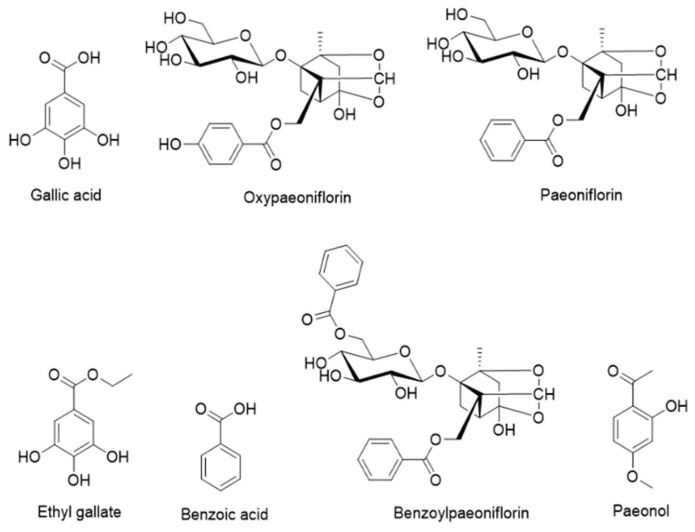
Chemical structures of the seven compounds of MRC analyzed in this study.

**Figure 3:**
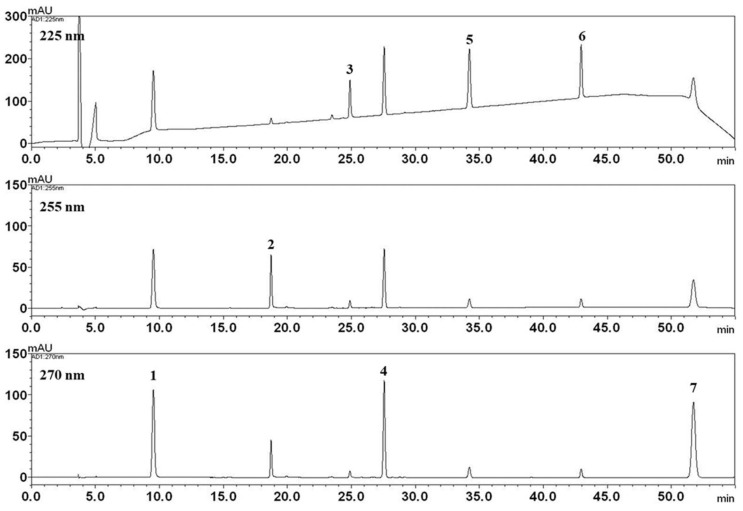
HPLC chromatogram of MRC analyzed by HPLC-PDA. Gallic acid (1), oxypaeoniflorin (2), paeoniflorin (3), ethyl gallate (4), benzoic acid (5), benzoylpaeoniflorin (6), paeonol (7).

Seven compounds were identified from the MRC extract and quantified: gallic acid, oxypaeoniflorin, paeoniflorin, ethyl gallate, benzoic acid, benzoylpaeoniflorin, and paeonol (Figure 2 and Figure 3). The quantity of each component was 2.442, 19.259, 39.938, 20.832, 2.212, 6.327, and 26.177 mg/g, respectively (Figure 2, Table 1).

### 3.2. Effect of MRC Identified Compounds on LPS-Induced NO Production in RAW 264.7 Cells

**Figure 4:**
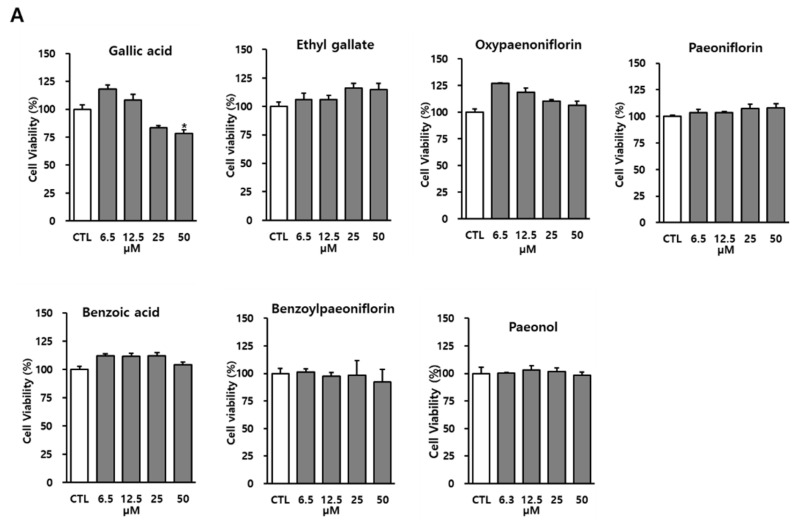

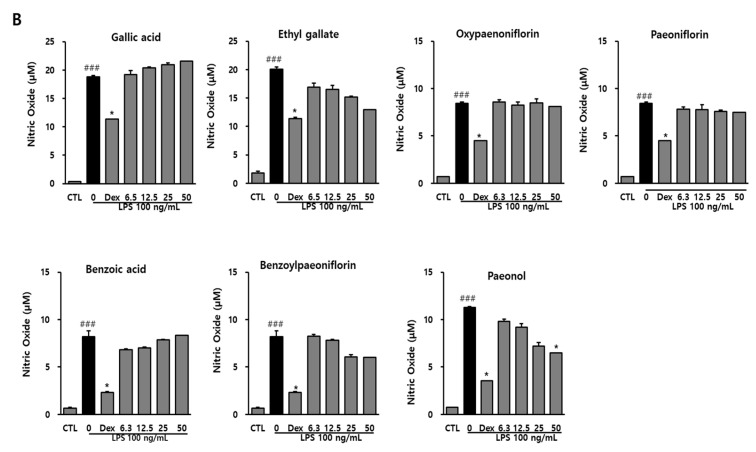
Effects of the MRC identified compounds on inhibition of nitric oxide (NO) production. RAW 264.7 cells were pretreated with various concentrations of seven compounds of MRC. (**A**) Comparison in cytotoxic effects of the seven compounds of MRC. (**B**) Comparison in NO production levels upon treatment with the seven compounds of MRC. Results are expressed as mean ± SEM (*n* = 3). Statistical significance was ### *p* < 0.001 compared to the non-treated group; * *p* < 0.05 compared to the LPS-treated group.

The anti-inflammatory effect of the MRC identified compounds was investigated by determining whether they elicit inhibitory effects on NO production in LPS-activated RAW 264.7 macrophages. In addition to evaluating cytotoxicity in RAW 264.7 cells, cell viability was shown no cytotoxicity at 50 μM except for gallic acid (Figure 4A). Gallic acid presented cellular toxicity at 50 μM (78.34 ± 3.14%). Based on the results of cell viability, treatment with ethyl gallate and paeonol resulted in a significant reduction in NO production in the LPS-activated RAW 264.7 cells (Figure 4B). Compared to those in the LPS-treated group (11.27 μM ± 0.40), the NO levels in the group treated with both paeonol (at concentrations of 6.3, 12.5, 25, and 50 μM) and LPS were significantly lower at 9.81 μM ± 0.26, 9.2 μM ± 0.33, 7.2 μM ± 0.43, and 6.47 μM ± 0.10, respectively (Figure 4). Similarly, ethyl gallate (25 and 50 μM) significantly reduced NO levels to 15.20 μM ± 0.10 and 12.23 μM ± 0.10, respectively, compared to those in the LPS-treated group (20.07 μM ± 0.37). These results suggest that ethyl gallate and paeonol may exert anti-inflammatory activities in LPS-activated RAW 264.7 cells. The cell viability results after treatment with ethyl gallate and paeonol suggest that the inhibitory effect on NO levels in LPS-activated RAW 264.7 cells was not due to cytotoxicity.

### 3.3. Effect of Paeonol on Pro-Inflammatory Cytokines IL-6 and TNF-α in Macrophages

**Figure 5:**
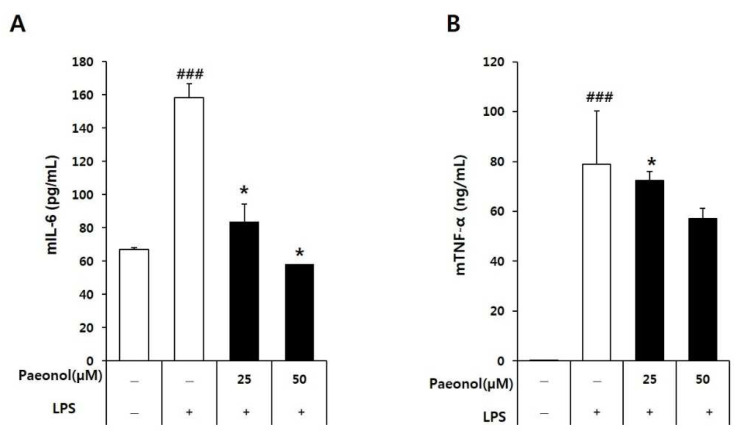
Effect of paeonol on the pro-inflammatory cytokines IL-6 (**A**) and TNF-α (**B**) in LPS-activated RAW264.7 macrophages. RAW264.7 cells were treated without or with paeonol for 2 h at 37 °C, before treatment with LPS for 22 h at 37 °C. The treated cell culture media were collected for cytokine determination. The absorbance values per treatment were used for the graph plots. Statistical significance was ### *p* < 0.001 vs. untreated group; and * *p* < 0.05 vs. LPS-alone group.

The effect of paeonol on cytokine production in LPS-activated RAW264.7 cells was determined by pretreating the cells with paeonol for 2 h and then treating them with LPS for 22 h. The cell supernatants were collected, and the cytokine levels were measured. Stimulation with LPS increased the levels of IL-6 and TNF-α compared with the control group. Treatment with paeonol inhibited the release of IL-6 and TNF-α in a concentration-dependent manner (Figure 5).

### 3.4. Effects of Paeonol on Inflammatory Cytokine mRNA Expression Levels in Macrophages

**Figure 6:**
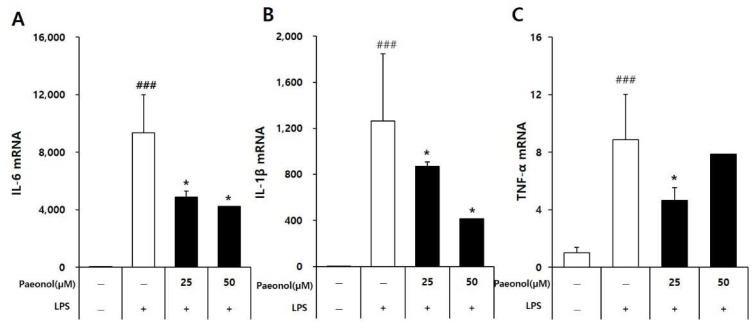
Paeonol decreases the mRNA expression of pro-inflammatory cytokines in LPS-activated RAW264.7 cells. The cells were treated with paeonol for 2 h, followed by LPS (100 ng/mL) stimulation for 4 h, respectively. After treatment, RNA was isolated from all groups and converted into cDNA, which was used for qPCR analysis. The relative expressions of IL-6 (**A**), IL-1β (**B**) and TNF-α (**C**) were calculated by normalizing with GAPDH gene expression. Statistical significance was ### *p* < 0.001 vs. untreated group; and * *p* < 0.01 vs. LPS-alone group.

The level of mRNA expression of pro-inflammatory cytokines, including IL-1β, IL-6, and TNFα, in RAW264.7 cells was analyzed using qPCR. As shown in Figure 6, the mRNA expression levels of IL-6 and IL-1β in the LPS-stimulated cells increased significantly compared to the control group, and this increase was reduced by paeonol in a concentration-dependent manner. These results confirm that paeonol inhibits some pro-inflammatory cytokine production at the transcription level.

### 3.5. Effects of Paeonol on LPS-Induced Expression of Mitogen-Activated Protein Kinase (MAPK) Proteins in Macrophages

**Figure 7:**
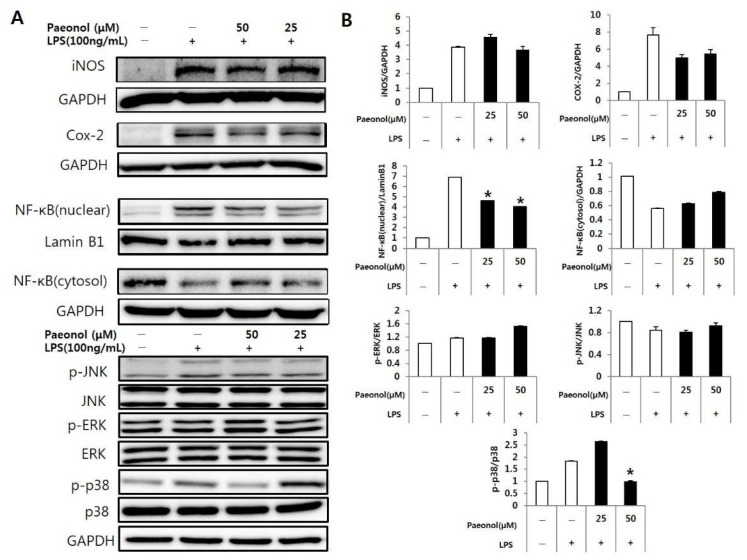
Inhibitory effects of paeonol on various MAPK protein expression levels in RAW264.7 cells. (**A**) The levels of p-JNK, p-ERK, p-p38, COX-2, iNOS, and NF-κB were determined using Western blotting. (**B**) Quantitative graph for western blots. The data represent the mean ± S.D of three experiments. * *p* < 0.05 compared to LPS alone.

RAW 264.7 cells were treated with paeonol, followed by stimulation with LPS. The expression levels of MAPK proteins were assessed by Western blotting. The levels of p-Jun kinase (JNK), p-extracellular signal-regulated kinase (ERK), p-p38, cyclooxygenase (COX)-2, and iNOS increased upon LPS treatment. The protein expression levels of NF-κB (nuclear) and COX-2 decreased significantly in the group treated with paeonol (Figure 7). The phosphorylation of p38 was significantly decreased in the high concentration (50 μM) of a paeonol treatment group. However, there were no changes in phosphorylation of JNK and ERK.

### 3.6. Reducing Effect of Paeonol on Inflammation in RAW 264.7 Cells

**Figure 8:**
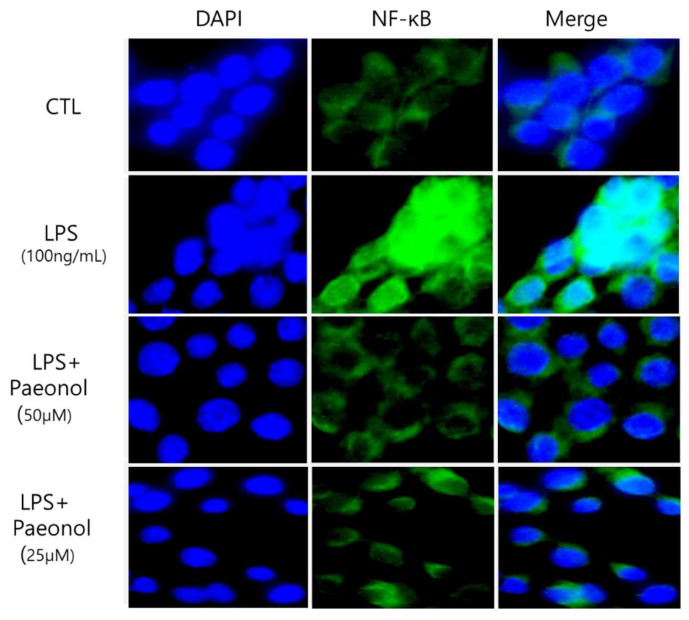
Effect of paeonol on pro-inflammatory NF-κB (nuclear) in LPS-activated RAW264.7 macrophages. RAW264.7 cells were pretreated without or with paeonol for 2 h at 37 °C. Then, the cells were treated with LPS for 22 h at 37 °C. LPS induced RAW264.7 cells were stained with NF-κB antibody (Alexa Fluor; green) for nuclear translocation and counterstained with DAPI (blue).

NF-κB is a transcription factor involved in proinflammatory responses. To determine the effect of paeonol on NF-κB (nuclear) in LPS-activated RAW264.7 cells, the cells were pretreated with paeonol for 2 h and then induced with LPS for 22 h. The cells were collected and analyzed for NF-κB protein expression. The levels of NF-κB (nuclear) increased in the LPS-treated than in the control group. NF-κB (nuclear) release was inhibited in a concentration-dependent manner in the paeonol-treated cells (Figure 8).

### 3.7. Effect of Paeonol on AAD Mice

**Figure 9:**
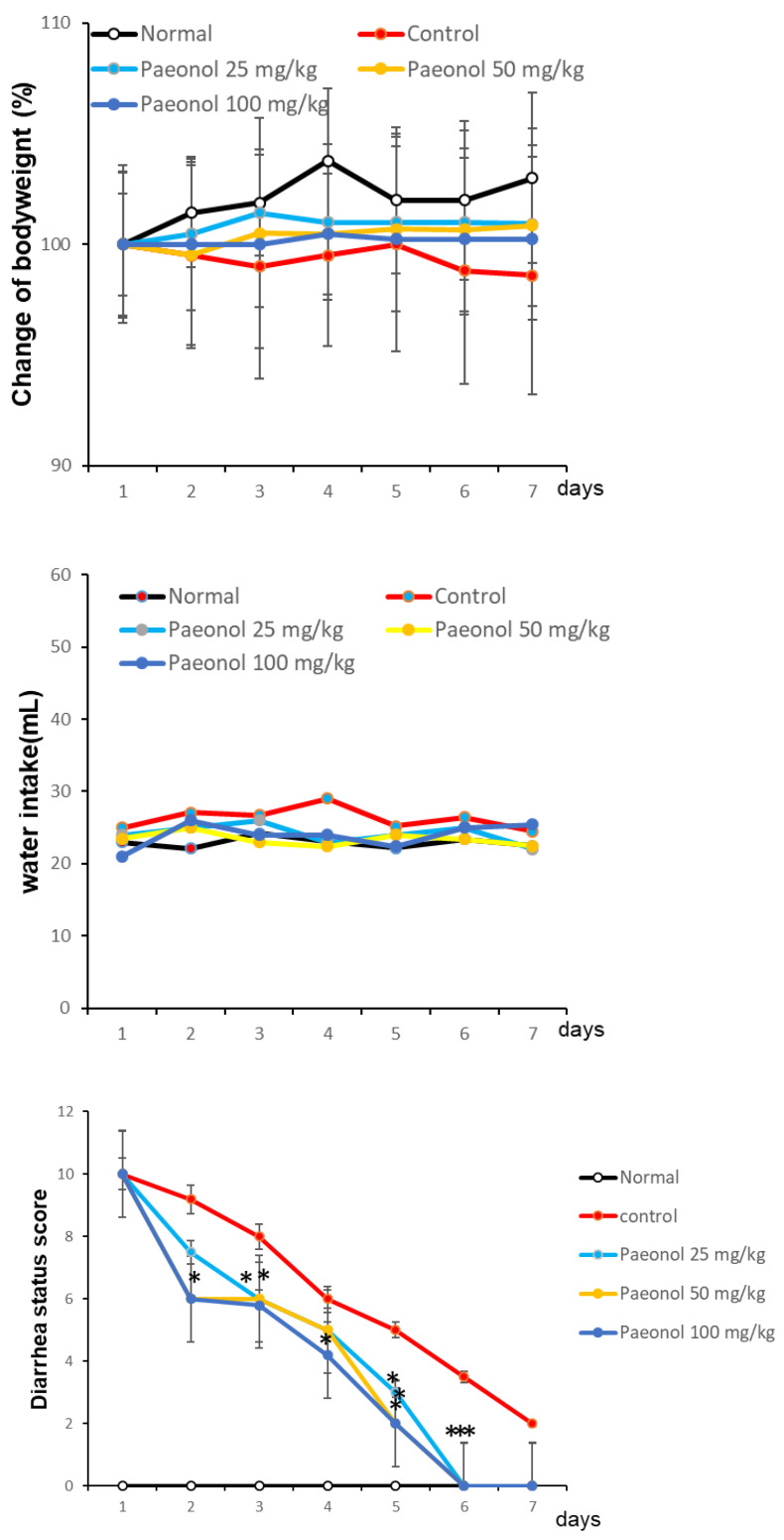
Effects of paeonol administration on body weight (**top**), water intake (**middle**), and diarrhea status (**bottom**).

After treatment with lincomycin hydrochloride, the animals presented with diarrheal symptoms, including increased frequency of defecation, reduced food consumption, and increased water intake. By the end of the experiment, the animals had 100% diarrhea, suggesting the successful establishment of the AAD mouse model. In the untreated group (normal group), the change in body weight was 100, 101.4, 101.8, and 103.7% on the 1st, 2nd, 3rd, and 4th day, respectively, and this change was minimal. In the control group that received the antibiotic, the weight change was 100, 99.5, 99.0, and 99.1% on the 1st, 2nd, 3rd, and 4th day, respectively; that is, weight in this group gradually decreased over time. The group administered with paeonol showed minimal change in body weight (Figure 9, top panel). The water intake of the untreated group (normal group) was 22.1, 24.2, and 23.1 mL on the 2nd, 3rd, and 4th days, respectively. The control group that received the antibiotic exhibited increased water intake compared to the untreated group (Figure 9, middle panel). There was no significant difference in water intake in the paeonol-administered group compared with the control group, although a tendency for decreased intake was noted. However, this difference was not significant (Figure 9, middle). The degree of diarrhea in the antibiotic-treated control group was 5.4, 10, 10, and 8 points, respectively, on days 2, 3, and 4, respectively. In the paeonol-administered group, recorded diarrhea scores were 7, 6, and 2.2 on the 2nd, 3rd, and 4th days, respectively; there was no significant difference among treatments with different concentrations; nonetheless, we observed a tendency for lower scores on successive days (Figure 9, bottom panel).

Normal group: group without treatment. Control group: AAD group. Paeonol 25, 50, 100 mg/kg group: after AAD was established, paeonol were administered orally. Values are expressed as mean (*n* = 5). Statistically significant values in the treatment groups compared with the control group were calculated by one-way ANOVA followed by Tukey’s test using R software version 3.3.3 compared to the control group; and * *p* < 0.05, ** *p* < 0.01, *** *p* < 0.005.

### 3.8. Effects of Paeonol on Inflammatory Cytokines IL-4 and TNF-α in AAD Mouse Serum

**Figure 10:**
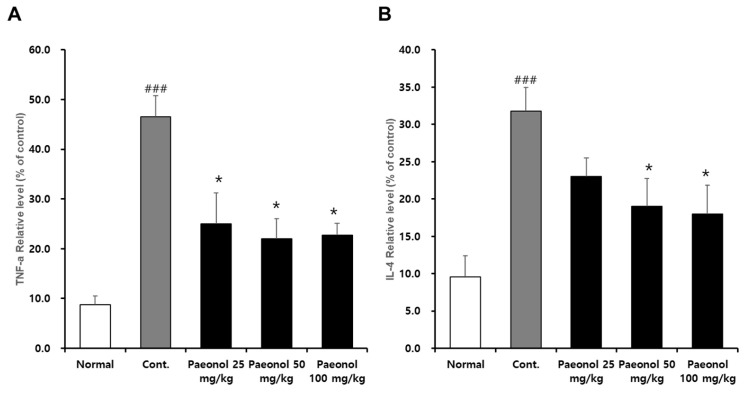
Effects of paeonol on TNF-α (**A**) and IL-4 (**B**) cytokine levels in a mouse model of lincomycin-induced diarrhea. Paeonol was orally administered to lincomycin-induced diarrhea mice. Blood was collected and used for the cytokine measurements. The absorbance values were used to generate the graph plots. ### *p* < 0.001 vs. normal group; and * *p* < 0.01 vs. cont. group.

To investigate the effect of paeonol on IL-4 and TNF-α levels in AAD mouse serum, blood was collected at the conclusion of the animal experiment. The levels of IL-4 and TNF-α were significantly increased in the control compared to the control, but the release of IL-4 and TNF-α was inhibited in a concentration-dependent manner in the paeonol-treated group (Figure 10).

## 4. Discussion

The major pathogen responsible for antibiotic-induced diarrhea is *C. difficile*, leading to CDAD; 10–20% of symptomatic patients are *C. difficile*-positive. A decrease in the ab-sorption function of the intestinal mucosa due to a deficiency of short-chain fatty acids can also cause diarrhea. Finally, antimicrobial treatments can lead to impaired defense against microorganisms in the colon and diarrhea [10,11,12]. Recommended treatments of CDAD are based on the administration of metronidazole or vancomycin; however, the recurrence rate is high. Contemporary research efforts are investigating the development of anti-inflammatory agents and treatment adjuvants that can effectively reduce various inflammatory mediators [4].

MRC is the dried root skin of Paeonia suffruticosa Andrew [13]; its properties contribute to overall good health and particularly gut health. MRC has anti-inflammatory properties and is effective in treating dysentery, inefficiency, and dyspepsia. Therefore, it is an important herbal medicine used broadly for its anti-inflammatory, antipyretic, and analgesic action [14]. Research on MRC includes animal experiments on anti-allergic activity [9], anti-inflammatory activity in human monocyte U937 cells [15], and animal experiments on anti-cancer activity [16]. MRC contains several biologically active compounds, including paeoniflorin, oxypaeoniflorin, benzoylpaeoniflorin, albiflorin, paeoniflorigenone, and pallotanin that do not bind to sugars [5]. Several studies have focused on the actions of individual active compounds of MRC, for example, on the anti-inflammatory effect of methyl gallate and the inhibitory effect of paeonol on arteriosclerosis [17,18]. In the present study, seven compounds were identified by the HPLC analysis of the MRC extract and quantified: gallic acid, oxypaeoniflorin, paeoniflorin, ethyl gallate, benzoic acid, benzoylpaeoniflorin, and paeonol. Our results on the evaluation of NO production showed that NO synthesis decreased in a concentration-dependent manner after the application of ethyl gallate and paeonol. NO is a neurotransmitter with multiple pathophysiological functions, including a role in vasodilation and regulation of blood pressure [19]. On the other hand, the NO produced can induce guanilate cyclase to the synthesis of cGMP from cGTP. These reactions cause the vasodilatation in the cardiovascular system and may cause a problem for patients with cardiovascular problems [20]. It is also involved in nonspecific immune responses and acts as a major mediator of inflammation and apoptosis in complex mechanisms such as tissue damage. In other words, NO is recognized as an immune regulator in the expression and resolution of inflammation; it directly or indirectly mediates inflammatory and infectious diseases and can have detrimental effects [21]. In this study, paeonol, one of the identified compounds in the MRC extract, showed the greatest inhibitory effect on NO synthesis, and was selected to investigate its potential anti-inflammatory action and alleviation effect of AAD symptoms.

AAD, a major symptom during antibiotic treatment, is associated with inflammation, and changes in intestinal structure. Thus, we confirmed the inflammation-related factors cytokines, iNOS, NO, Cox-2, MAPKs, and NF-kB in Raw 264.7 cells. The changes in cyto-kines may also reflect the host’s inflammatory status as it relates to antibiotic therapy, such as interleukins and tumor necrosis factor, which play important roles in the immune system as communicators between immune cells. An inverse relationship has been re-ported between IL-1β, IL-4 and TNF-α levels and grain fiber intake [22]. IL-4 and TNF-α are widely used markers in AAD experiments and in some cases not used for other inflammatory diseases. However, this may be a difference in disease [23,24]. In addition, IL-4 is a cytokine that is greatly increased and well detected in AAD [24]. The in vitro studies showed that paeonol effectively reduced the mRNA expression levels of IL-6, IL-1β, and TNF-α, which are known indicators of inflammation. Among several cytokines related to immunity and inflammation, IL-1β, IL-6, and TNF-α are representative inflammatory cy-tokines produced by macrophages, and their excess production leads to pathological con-sequences. IL-6 is mainly secreted by B and T cells and monocytes, promotes the prolifera-tion and differentiation of B and T cells, and exhibits its functions in various systems. It is a key factor in response and host defense mechanisms and has a regulatory role in the hematopoietic system [25,26]. TNF-α is present in high concentrations at the site of in-flammation and has been studied for a long time as a pathological cause of various dis-eases and conditions, including sepsis, cancer, rheumatoid arthritis, ulcerative colitis, and Crohn’s disease [27,28,29,30]. COX is an essential enzyme for converting arachidonic acid to prostaglandin. COX inhibitors can inhibit the production of pro-inflammatory prosta-glandins at the site of inflammation. COX-1 is expressed in most tissues of the human body and is involved in normal physiological functions, whereas COX-2 is secreted in large amounts in pathological environments such as inflammation and promotes tumor-associated responses and resistance to apoptosis [31]. However, exposure to non-steroidal anti-inflammatory drugs (NSAIDs) is known to increase substantially the risk of upper gastrointestinal bleeding and perforation (UGIB) [32]. The development of COX-2 inhibitors that can reduce some of the side effects of existing non-steroidal anti-inflammatory drugs (NSAIDs) is a recent promising advancement in managing inflammation. In particular, the search for products of natural origin is timely and supported by a growing number of studies on the influence of herbal medicines on inhibiting COX-2 activity [33]. In the present study, it was observed that paeonol had an effective anti-inflammatory action by inhibiting COX-2.

NF-κB (nuclear) regulates various physiological processes, including cell cycle and survival and immune and acute responses. In macrophages, the production of NF-κB (nu-clear) was inhibited by paeonol. NF-κB induces the transcription of inflammatory media-tor genes as it moves into the nucleus after disassociation with the NF-κB inhibitor I-κB. Since NF-κB induces the gene expression of inflammatory cytokines, junction molecules important for immune cell migration, and chemokines, treatment with paeonol can re-duce the production of inflammatory mediators.

Pathways that transmit signals from the cell membrane to the nuclear MAPK, a fac-tor acting on the MAPK pathway, are responsible for various functions such as cell prolif-eration, differentiation, death, and regulation of cellular responses to cytokine stress; MAPKs are commonly classified into three families: ERK, JNK, and p38 [31]. Interestingly, phosphorylated p38 increased at low concentration (25 μM) of paeonol but decreased when its concentration was higher (50 μM); this suggests that paeonol has a potential an-ti-inflammatory effect at high concentration.

Overall, the above in vitro results confirmed that paeonol has an anti-inflammatory property by reducing the secretion of inflammatory me-diators. In the in vivo study, a mouse model of diarrhea was successfully developed by administering lincomycin. In this model, it was verified that the symptoms of diarrhea improved after treatment with paeonol at all concentrations (25, 50, and 100 mg/kg) compared to those of the control group. It was further confirmed that the expression levels of TNF-α and IL-4 in the mouse serum effectively decreased at all concentrations of paeonol (25, 50, and 100 mg/kg). Together, these results suggest that paeonol is effective in reducing inflammatory substances caused by lincomycin-induced diarrhea.

As such, efficacy in reducing the symptoms of antibiotic-induced diarrhea and the levels of inflammatory mediators is thought to be correlated with the efficacy of paeonol administration. In the present study, it was observed that the application of paeonol, an active compound of MRC, improved diarrheal symptoms induced by antibiotics and low-ered inflammatory responses.

## 5. Conclusions

As a result of this study, it was observed that the use of paeonol improved the symptoms induced by antibiotics and lowered the inflammatory response in mice with diarrhea induced by lincomycin administration. In this study, a total of seven compounds were identified and quantified from MRC, including gallic acid, oxypaeoniflorin, ethyl gallate, benzoic acid, benzoylpaeoniflorin, and paeonol. As a result, benzoylpaeoniflorin, paeonol, and ethyl gallate showed the highest content in the order. All seven compounds of MRC did not change the cell viability, and among them, it was confirmed that ethyl gallate and paeonol decreased the production of nitric oxide. Paeonol reduced IL-6 and TNF-α in a concentration-dependent manner in RAW 264.7 rat macrophages, was effective in reducing the mRNA expression levels of IL-6 and TNF-α, and inhibited COX-2 and NF-κB. From the above results, it is thought that paeonol, an active compound of MRC, can be used as a useful substance in the treatment of antibiotic-induced diarrhea by improving the symptoms of antibiotic-induced diarrhea and reducing inflammation mediators. Other components of the MRC extract could contribute to its known anti-inflammatory and antibacterial activity and should be tested for their possible activity.

## Figures and Tables

**Table 1:** Linear range, regression equation, and coefficient of determination (r^2^) for marker compounds.

Compound	Detection (nm)	Linear Range (μg/mL)	Regression Equation	r^2^
Gallic acid	270	1.56–100.00	y = 52178.41 x − 10097.10	0.9996
Oxypaeoniflorin	255	1.56–100.00	y = 11397.17 x − 3278.35	0.9996
Paeoniflorin	225	1.56–100.00	y = 17069.03 x − 6010.13	0.9995
Ethyl gallate	270	0.78–50.00	y = 60206.20 x − 594.86	0.9996
Benzoic acid	225	1.56–100.00	y = 73790.50 x + 33617.21	0.9997

## Data Availability

Not applicable.
